# Fenestrated pedicle screws for cement-augmented purchase in patients with bone softening: a review of 21 cases

**DOI:** 10.1007/s10195-011-0164-9

**Published:** 2011-11-08

**Authors:** Luca Amendola, Alessandro Gasbarrini, Matteo Fosco, Christiano Esteves Simoes, Silvia Terzi, Federico De Iure, Stefano Boriani

**Affiliations:** 1 Department of Orthopedics and Traumatology, Spine Surgery, Ospedale Maggiore “C.A. Pizzardi”, Largo Nigrisoli 2, Bologna, Italy; 2 Department of Orthopaedic Surgery, Istituti Ortopedici Rizzoli, Bologna, Italy; 3 Department of Orthopedics and Traumatology, Spine Surgery Unit, Hospital Felício Rocho, Belo Horizonte, Brazil; 4 Department of Oncological and Degenerative Spine Surgery, Istituti Ortopedici Rizzoli, Bologna, Italy

**Keywords:** Fenestrated pedicle screw, Polymethylmethacrylate, Osteoporotic bone, Spine tumor

## Abstract

**Background:**

This prospective mixed cohort study was designed to evaluate the middle- to long-term purchase of cement-augmented pedicular screws in patients with poor bone quality. The growing number of surgical procedures performed in the spine has highlighted the problem of screws loosening in patients with poor bone stock due to osteoporosis and/or tumors. Different methods of increasing screw purchase have been reported in the literature, including polymethylmethacrylate (PMMA) augmentation.

**Materials and methods:**

From September 2006 to April 2008, 21 patients with a poor bone stock condition due to osteoporosis or tumor underwent posterior stabilization by fenestrated pedicle screws and PMMA augmentation. Pain improvement and long-term clinical outcome were assessed by visual analogue scale (VAS) score and SF-36 health survey (SF-36) questionnaire. Implant stability was evaluated by plain radiography and CT scans performed three days after surgery and every three months thereafter. After the first 12 months, radiologic controls were taken once a year in all surviving patients. Complications were evaluated in all cases.

**Results:**

All patients were clinically and radiographically followed up for a mean of 36 months. VAS scores and SF-36 questionnaires showed a statistically significant reduction in pain and improvement in the quality of life. No radiological loosening or pulling out of screws was observed. In two cases, cement leakage occurred intraoperatively: one patient who suffered from a transitory nerve root palsy improved spontaneously, while the surgeon immediately removed the excess cement before setting in the other case. In three cases, the post-op CT scan revealed a small amount of cement in the canal without clinical relevance.

**Conclusions:**

Fenestrated screws for cement augmentation provided effective and lasting purchase in patients with poor bone quality due to osteoporosis or tumors. No case of loosening was recorded after a mean follow-up of 36 months. The only clinical complication strictly related to PMMA screw augmentation did not require further surgery.

## Introduction

The use of pedicle screws for spine stabilization in the elderly is increasing, as their use enables a fast functional recovery under different conditions, such as fractures, deformities, infections, and tumors. On the other hand, mechanical failures due to screw loosening are becoming a major cause of morbidity in the elderly because of their poor bone quality. Many solutions have been proposed to reduce this risk, including the use of expandable screws [1], hydroxyapatite-coated screws [2, 3], bicortical screw purchase [4], larger diameter screws [1, 5, 6], and polymethylmethacrylate (PMMA) augmentation [7, 8].

An improvement in PMMA augmentation procedures can be achieved through the use of fenestrated pedicle screws specifically designed for cement injection. Once PMMA has been extruded though the screw holes, it sets due to polymerization, creating a continuous mass between the core of the screw and the cancellous bone in the vertebral body.

The aim of this single-center observational study was to evaluate the middle- to long-term performance of cement-augmented fenestrated pedicle screws in patients with bone softening caused by osteoporosis and/or neoplastic diseases.

## Materials and methods

From September 2006 to April 2008, 201 surgical procedures were performed by means of a posterior approach using pedicle screws in the thoracic and lumbar spine for the treatment of traumatic, degenerative, or neoplastic conditions.

In 21 patients with bone softening caused by osteoporosis or neoplastic conditions, fenestrated screws were used for cement augmentation in order to achieve better purchase (Table 1). There were 11 women and 10 men, with a mean age of 67.2 years (SD = 9.1; range 55–85).

**Table 1:** Patient data

Patient no.	Sex	Age	Disease	Surgery date	Cemented screw	Uncemented screw	FU period (mos)	Note
1	M	66	Degenerative disease	12/09/2006	1	9	52	
2	F	76	Tumor (myeloma)	11/12/2006	4	0	48	Cauda syndrome
3	F	68	Post-traumatic kyphosis	17/01/2007	2	7	48	
4	M	73	Tumor (prostate)	24/02/2007	4	0	13	DOD
5	M	85	Fracture	10/05/2007	4	8	44	Superficial infections, deep vein thrombosis
6	F	57	Failure of previous surgery	25/05/2007	4	6	43	
7	F	75	Failure of previous surgery	05/06/2007	1	11	42	
8	F	56	Degenerative disease	08/06/2007	7	5	41	Surgical revision
9	M	57	Failure of previous surgery	11/06/2007	10	8	40	Superficial infections, transient cerebral ischemia
10	M	62	Tumor (prostate)	13/06/2007	4	2	36	
11	M	70	Failure of previous surgery	17/08/2007	2	10	38	Cement leakage
12	F	76	Tumor (hypernephroma)	31/08/2007	4	4	36	Cement leakage
13	F	77	Fracture	22/09/2007	4	0	40	
14	F	58	Tumor (breast)	09/11/2007	3	4	24	DOD
15	F	79	Tumor (breast)	07/02/2008	4	2	34	
16	F	70	Tumor (breast)	13/02/2008	4	4	34	
17	M	72	Post-traumatic kyphosis	27/02/2008	4	6	36	System removal
18	M	55	Tumor (myeloma)	13/03/2008	4	0	33	
19	M	55	Tumor (myeloma)	31/03/2008	4	0	30	
20	M	58	Failure of previous surgery	09/04/2008	2	8	32	
21	F	66	Tumor (hypernephroma)	14/04/2008	5	0	21	DOD

Indication for the use of cemented screws was confirmed by evaluating the degree of osteoporosis in all patients. *T* score ≤ 2.5 SD was an indication for this technique [9], and it was found in two patients with degenerative disease, two with traumatic fracture, two with post-traumatic kyphosis, five cases of failed previous surgery, and ten neoplastic patients (three myeloma, seven metastases).

A total of 81 fenestrated screws were implanted (min 1; max 10), always in combination with standard screws (a total of 88 standard screws were implanted) of the Legacy system (Medtronic, Tolochenaz, Switzerland). In tumor patients, we performed short fixations without fusion, one or two levels below and above the lesion.

All patients provided their informed consent for surgery. The study was approved by the local ethical committee, and performed in accordance with the ethical standards of the 1964 Declaration of Helsinki as revised in 2000. Patients were carefully followed up through periodic clinical and radiologic examinations. In all cases, pre- and postoperative clinical details were collected: pain intensity was evaluated by VAS score and quality of life by SF-36 questionnaire.

Implant stability was evaluated by plain radiography and CT scans performed three days after surgery and every three months thereafter. After the first 12 months, radiologic controls were taken once a year in all surviving patients. Complications were evaluated in all cases.

Radiographic evaluation at follow-up included loss of sagittal alignment (kyphosis). Standard radiograms were also used to assess how the fenestrated screws supported the bone fusion: the presence of trabecular bone bridging the interspace between the adjacent vertebral bodies. This bone fusion evaluation did not include neoplastic patients, as bone grafting is seldom used, and fusion is difficult or impossible to achieve because of the side effects of perioperative radiotherapy, chemotherapy, steroid drugs, malnutrition, and anemia [10]. Intra- and postoperative complications were also recorded.

### Surgical technique

**Fig. 1:**
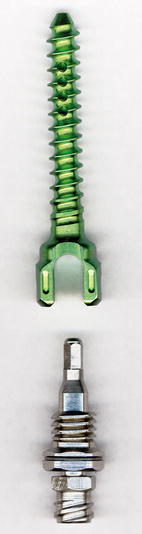
Fenestrated cannulated pedicle screw

**Fig. 2:**
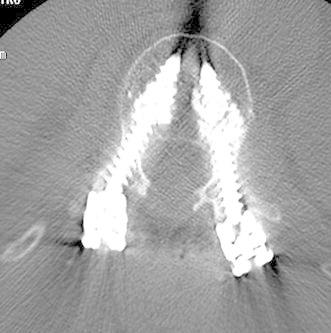
Postoperative CT scan showing the cement extruded around the screws

The titanium screws used in this study have a cannulated core and are fenestrated with two series of three holes set into the grooves of the distal portion of the thread. They are available in diameters of 6.5 and 5.5, and as both monoaxial and polyaxial uploading models (Fig. 1). The cement, injected under pressure through the cannulation, is extruded through the holes to fill the spaces inside the osteoporotic cancellous bone, thereby increasing the purchase of the screw (Fig. 2).

**Fig. 3:**
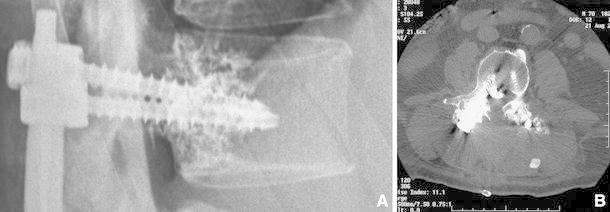
**a** Screw too short: risk of epidural leakage. **b** Epidural leakage of the cement

The fenestrated screw is inserted into the pedicle, as done with conventional screws. The length of the screw (Fig. 3a) and the positions of the holes, located as far as possible from the posterior wall, must be carefully checked in order to prevent possible leakage into the canal (Fig. 3b). The screw and the cement injector are connected by a specifically designed connector. Common vertebroplasty cement can be delivered through its specific gun. The amount of cement injected into each screw varies from 1.5 to 3 cc. With experience, we found that the ideal amount of cement to inject was 2 cc. PMMA is always injected under continuous image intensifier visualization.

The rod (5.5 diameter) must only be connected to the screws once the polymerization process has been completed, in order to prevent microfractures at the screw/cement/bone interface.

The integrity of both the anterior and the posterior wall of the vertebral body was verified by CT scan in all patients to prevent both retroperitoneal and epidural leakage.

### Statistical analysis

Data collection and analysis were performed using SPSS version 15.0. Data are reported as the mean, the standard deviation (SD), and the range if continuous, and as the absolute and relative frequencies if categorical. Pre- and postoperative VAS scale scores and SF-36 results were compared using the Wilcoxon nonparametric test for paired samples with a level of significance of 0.05.

## Results

Patients were observed, via clinical and radiological examinations, for a minimum of 30 months or until death (which occurred in three cases, at 13, 21, and 24 months, respectively). The mean follow-up time was 36.4 months (SD = 9.3; range 13–52).

Pain was the most common complaint before surgery, with a mean VAS of 8.2 (SD = 0.7; range 7–10). However, limping and lower limb weakness were the main indications for surgery in ten patients. Ten patients affected by tumors were unable to stand because of mechanical incompetence due to neoplastic bone erosion.

Walking ability improved dramatically in all patients complaining of claudicatio spinalis before surgery. All neoplastic patients were able to stand and to walk after surgery.

Surgery was associated with a significant decrease in VAS score (*n* = 21, *Z* = − 4.040, *P* < 0.001), and pain intensity improved significantly (Table 2), with a mean VAS score of 1.7 (SD = 1.5; range 0–6) recorded during the last clinical control.

**Table 2:** Test statistics

	Pre-op	FU	*Z* ^a^	*P*
SF-36				
Physical functioning	18.1	66.9	−4.022	<0.001
Role-physical	9.5	52.4	−3.874	<0.001
Bodily pain	17.6	61.5	−4.039	<0.001
General health	25.2	63.7	−4.020	<0.001
Vitality	33.1	65.7	−4.025	<0.001
Social functioning	33	66.4	−4.033	<0.001
Role-emotional	3.1	55.1	−3.891	<0.001
Mental health	51.6	76.6	−4.024	<0.001
VAS	8.2	1.7	−4.040	<0.001

Wilcoxon signed-rank test

^a^Based on positive ranks

The comparison of preoperative SF-36 results and those at final follow-up also showed a statistically significant improvement in the quality of life (Table 2).

**Fig. 4:**
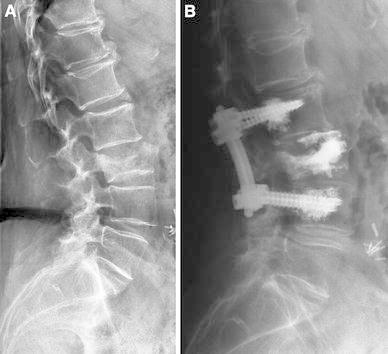
M.B., 77 years. **a** Osteoporotic fracture of L3. VAS: 9. Unable to stand or walk. **b** Short fixation with fenestrated screws and cement augmentation. Vertebroplasty of L3. Immediate recovery of function. 40-month follow-up: VAS: 1. Able to walk without support

No cases of loosening or pulling out of screws were recorded. The balance achieved by surgery was never lost, despite the poor bone condition (Fig. 4): the mean loss of sagittal correction at final follow-up compared to the postoperative one was 4° (SD = 3; range 0–10).

**Fig. 5:**
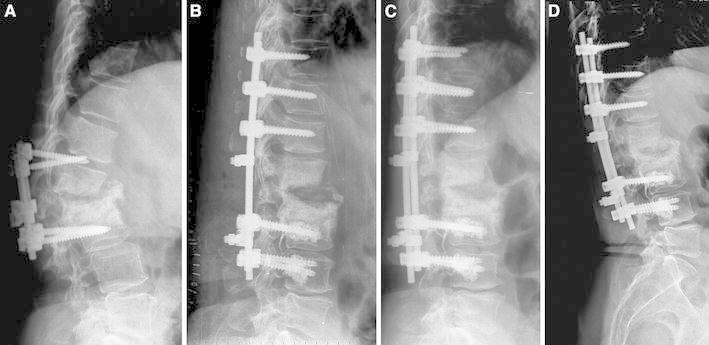
P.A., 57 years. **a** Failure of previous surgery performed in another hospital. **b** Postoperative X-ray. **c**, **d** Follow-up X-rays taken three and six months after treatment, respectively

Bone fusion was achieved in all non-cancer patients within six months (Fig. 5), with no cases of pseudoarthrosis being recorded.

Complications are summarized in Table 1. One patient underwent surgical revision to treat an adjacent vertebral body fracture following a car accident injury. Even after the traumatic event, no displacement or loosening of the construct occurred.

PMMA-related complications were found in five cases. In two patients, cement leakage was noticed intraoperatively. In both, 3 cc of cement had been injected. A nerve root palsy was the clinical consequence in one case. Cement was not removed, as it was found to be already solid (Fig. 3b). Following a rehabilitation program, this patient partially recovered his walking ability (follow-up 14 months). In the other patient, the surgeon removed the excess cement during the same surgical procedure without neurologic sequelae. In all other cases, a maximum of 2 cc per screw were injected, and no major leakage into the canal occurred. In three of these, a small amount of epidural cement was found on postoperative CT scan, but without clinical relevance.

There were two early postoperative superficial infections that were successfully treated by antibiotic therapy. One of these patients was found with a lower limb venous thrombosis seven days after surgery. The other one suffered a transient cerebral ischemia three days after surgery.

One patient had a cauda syndrome due to a postsurgical hematoma that appeared on the third day. This patient, who suffered from heart disease, probably resumed oral anticoagulant therapy too early. She underwent urgent surgical revision, with drainage, debridement, and widening of the laminectomy. Neurological function slowly recovered until it was completely normal.

One more patient fell off a ladder, suffering fractures of the proximal and distal anchorage vertebrae, resulting in implant mobilization. Due to the patient’s poor general condition, we simply carried out implant removal and vertebroplasty of the injured vertebrae.

Finally, three patients with metastases died of the disease 13, 21, and 24 months after treatment, respectively.

## Discussion

It is well known that an age-related reduction in bone density reduces the mechanical properties of the bone–screw interface. Enlarging the spaces in the trabecular meshwork limits the immediate mechanical grip of the screws and compromises integration at the interface between bone and metal, thereby facilitating loosening of the implant. Surgical treatment of the osteoporotic vertebral column is therefore burdened with a high incidence of implant failure due to pedicle screws loosening as a result of pull-out phenomena [4, 11–13].

Similar conditions can be found during revision surgery of a previous implant or whenever a local or a systemic disease causes a deterioration in bone quality.

Various technical strategies for improving pedicle screw grip have been described in the literature [1–8].

The use of screws with a larger diameter than those previously implanted proved to be effective in revision surgery; they had to be at least 2 mm larger to ensure reliable purchase [5]. Nevertheless, it is not always possible to use bigger screws for anatomical reasons. Moreover, their use increases the risk of fracture of the pedicle [1, 6].

The use of longer screws, anchoring into the anterior cortex of the vertebral body, has also been proposed. Upon using this type of fixation, Zindrick et al. [4] found that the force required to loosen the screws increased by 30%. On the other hand, the risk of vascular or visceral injury cannot be ignored.

Expansion screws have also been used. The anterior two-thirds of this type of screw expands in diameter once the screw has passed through the pedicle. Experimental results in osteoporotic bone [14] have shown that such screws are more resistant to pull-out. In 2001, Cook et al. [1] published their case review of 145 patients in whom expansion screws had been used in the presence of osteoporosis for implant revision and sacral anchorage; their clinical results were comparable to those obtained by means of a conventional technique in unselected patients.

Coating pedicle screws with hydroxyapatite can also improve implant stability. In ovariectomized sheep, coated screws displayed significantly greater resistance to extractive torque stress [2]. In addition, in an experimental canine model, Hasegawa et al. [3] found that hydroxyapatite-coated screws offered 1.6-fold greater resistance to pull-out stresses than uncoated titanium screws. Nevertheless, bone/screw interface integration is not expected to happen immediately, so primary stability does not differ much from that of standard screws.

The use of PMMA to fill and stabilize implants has been a standard procedure in orthopedic surgery for decades. More recently, however, due to the popularity of kyphoplasty and vertebroplasty, the use of PMMA in spine surgery has become common. Indeed, PMMA can also be used to reinforce pedicular fixation in cases of impaired bone quality. Several experimental and clinical studies have proven that PMMA augmentation is capable of improving resistance to pull-out in osteoporotic and normal vertebrae [7, 8, 15–20]. In poor-quality bone, a gap is frequently created between the threaded portion of the screw and the trabecular spongy bone; cement strengthens the bone/metal interface at such points. PMMA screw augmentation may increase both the primary stability and the fatigue resistance of the implants [7, 8], making them better able to withstand the axial stresses responsible for pull-out [15–19].

PMMA reinforcement of pedicle screws can be carried out by first injecting the cement into the pedicle and subsequently inserting the screw. This technique, however, risks increasing the pressure inside the borehole, which may cause leakage of the cement, with possible embolism in the venous plexuses or cord damage.

More recently, fenestrated screws through which acrylic or biological cement can be injected have been placed on the market [21]. In 2005, Yazu et al. published an experimental study conducted on osteoporotic vertebrae from cadavers; these authors compared the performance of fenestrated screws with that of traditional screws [20]. Cement injection can be modulated more accurately using fenestrated screws, reducing the risk of leakage into the canal and/or foramina.

In one of the patients reported here, such leakage occurred, causing transitory nerve root palsy. In this case, however, an excessive amount of cement (>3 cc) had been injected. This complication was probably due to our limited experience with this technique, as we were at the beginning of the learning curve. In the other case, in which an initial leakage of cement was seen, its insertion was promptly interrupted; adequate grip was nevertheless achieved, and the postoperative course was uneventful.

To avoid this complication, it is mandatory to carefully evaluate the integrity of the base of the pedicle on CT scan. This technique is strongly contraindicated whenever a breach is detected in the posterior wall or pedicle [21]. The length of the screw should be such that the fenestration can be positioned in the anterior portion of the vertebral body. No more than 2 cc of PMMA should be injected under strict, continuous fluoroscopic monitoring, ceasing injection if leakage is observed. Screw insertion should be carried out precisely inside the pedicle, as breaching its medial cortex may allow the cement to leak into the epidural space.

PMMA injection through fenestrated cannulated screws provided additional stability in fixation procedures carried out on osteoporotic vertebral columns, leading to good pain control in all patients. In tumor patients, this additional stability allowed shorter constructs to be performed, reducing morbidity and preserving the mobility of the adjacent segments, all without increasing the risk of failure. Moreover, cement-augmented screws did not seem to affect fusion in osteoporotic patients with different spine pathologies.

No screw loosening was recorded after a mean follow-up of 36 months. Although the results in this series of 21 patients treated at the same center were positive, this technique should be validated in a larger series of patients.
